# Numerical Simulation and Experimental Study of the Extrusion Process in Additive Manufacturing for High-Viscosity and High-Solid-Content Multi-Component Energetic Materials

**DOI:** 10.3390/mi17020172

**Published:** 2026-01-28

**Authors:** Dashun Zhang, Shijun Ji, Ji Zhao, Juan Du, Handa Dai, Suhui Sun, Ke Guo

**Affiliations:** 1 School of Mechanical and Aerospace Engineering, Jilin University, Changchun 130012, China; zhangdashun666@126.com (D.Z.); jzhao@jlu.edu.cn (J.Z.); dhd@jlu.edu.cn (H.D.); 2 Changchun Equipment and Technology Research Institute, Changchun 130012, China; 18643113563@163.com (J.D.); 13159615568@163.com (S.S.); sunsuhui818@126.com (K.G.)

**Keywords:** additive manufacturing, high viscosity and high solid content, numerical simulation, experimental verification

## Abstract

A combined numerical simulation and experimental validation approach was employed to investigate the phenomena of screw adhesion and nozzle clogging, which occur frequently during material conveying and extrusion of high-viscosity, high-solid-content multi-component energetic materials in additive manufacturing. First, conical and cylindrical screws were designed. Through simulation calculations of the energetic material extrusion process, patterns in the variation in internal pressure and shear rate within the screw were analyzed, providing guidance for the design of the printing equipment. Second, a Z-shaped stirring paddle kneading device and a dual-nozzle printing device featuring horizontally and vertically arranged two-stage screws were designed. Through extrusion experiments with PBX (polymer-bonded explosive) slurry, the optimal matching relationship between the kneading rate and the extrusion rates of the horizontal and vertical screws was obtained. Finally, additive manufacturing of complex-shaped PBX charges using high-viscosity energetic materials was successfully accomplished. This confirms the further optimization of the additive manufacturing equipment in terms of safety control, precision control, and adaptability to complex structures under extreme operating conditions. The results indicate that the cylindrical screw outperforms the conical screw, and with a screw clearance of 3mm, it represents the optimal design solution. During the kneading process, a screw rotational speed of 25 rpm was used. After kneading for 3 h, the slurry exhibited good uniformity, with a solid content of approximately 70% and relatively small deviation. During the extrusion process, a nozzle diameter of 1.55 mm combined with a rotational speed of 5 rpm for the horizontal screw (feeding screw) and 7 rpm for the vertical screw (extrusion screw) can satisfy the requirements of the “starved feeding” mode, thus achieving continuous and stable filament formation of the slurry.

## 1. Introduction

Energetic materials, as a core energy source, are widely used in military, aerospace, engineering blasting, and other applications [1]. Traditional charge-forming processes suffer from issues such as long loading times, uneven charge density, and weak bonding between explosive strands [2]. With the advancement of additive manufacturing technology [3], breakthroughs have been made in overcoming the structural limitations of conventional manufacturing [4], enhancing design flexibility, and optimizing material utilization and performance consistency [5]. This significantly improves the R&D efficiency, reliability, and production safety of energetic charges. Particularly in demanding fields such as military and aerospace, where high precision and customization are critical, additive manufacturing demonstrates advanced technological advantages. It enables the production of complex-shaped composite explosives, meeting the urgent developmental needs of weapon systems for energetic materials with “controllable propulsion and adjustable lethality”.

However, the high viscosity and high solid content (wt.%) of energetic materials pose significant challenges in additive manufacturing, including clogging during conveying, unstable extrusion [6], nozzle wear/blockage, temperature fluctuations, and compromised material homogeneity. These issues not only degrade printing accuracy and efficiency but may also trigger safety hazards due to the sensitive nature of the materials. Consequently, specialized equipment and process optimizations are imperative. Innovative approaches within the field, such as a novel screw-pressing charging method, have been developed to improve automation and uniformity in the melt-casting of polymer-based energetics, utilizing simulation frameworks to guide safe and efficient equipment design [7]. Furthermore, insights from the processing of other high-solid-loading systems are valuable. For instance, studies on preparing alumina suspensions for microfabrication demonstrate that achieving homogeneous, high-solid-loading (e.g., 60 wt.%) slurries suitable for precision molding hinges on the optimization of dispersants and binder systems [8]. Currently, there is a lack of mechanistic studies addressing the multi-physics coupling (fluid–thermal–mechanical–chemical interactions) [9], multi-scale behavior (microscopic particles to macroscopic structures), and multi-performance coupling (formability–mechanical properties–combustion–safety) of energetic materials. Moreover, few theories can effectively guide industrial applications, resulting in a disconnect between theoretical research and practical implementation.

In recent years, with the rapid development of computer hardware and commercial computational fluid dynamics (CFD), numerical simulation has become a pivotal tool for studying the AM process of energetic materials. Researchers have employed simulations to dissect the complexities of extrusion-based printing, a key technique for solid propellants. Comparative studies between screw and plunger extrusion mechanisms reveal their distinct flow characteristics: plunger systems offer more stable flow and uniform output, while screw systems generate complex rheology including backflow but operate at lower average pressure, with both capable of achieving comparable final printing quality [10]. Zong et al. [11] reviewed the current status of the 3D printing field, analyzed the relevant mechanisms, and provided guidance for future research. Dunju, Wang et al. [12] designed and printed CL-20/HTPB 3D-printable explosive ink, optimizing ink uniformity by adjusting the CL-20 concentration and the ratio of binder to curing agent, which achieved high-quality continuous printing and provided a reference for the mixing ratio of slurry and binder in this study. Yang et al. prepared a new type of energetic oligomer and used the composite propellant composed of it and CL-20 for SLA 3D printing [13]. Compared with inert binders, this propellant showed a 15% increase in thermodynamic energy and a 480% increase in burning rate under 100 MPa pressure. Zhang et al. introduced energetic binders into the photocurable 3D printing system and realized the printing of microstructures using UV-assisted direct writing technology, providing a basis for the molding of explosive columns in this study [14]. However, due to the high viscosity of energetic materials [15] (zero shear viscosity: 46249; limiting shear viscosity: 1.507), whether the screw can smoothly convey and extrude energetic materials while ensuring safety remains a crucial factor in guaranteeing the quality of molded parts and processing efficiency. Therefore, this study used ANSYS 2022 R1/FLOTRAN software to simulate and calculate the influence of screw clearance changes on pressure and shear rate during the printing of conical and cylindrical screws [16]. The kneading process and the optimal matching speed of the cross-longitudinal two-stage screws were systematically and intuitively analyzed, identifying the “starvation feeding” rule, in which the transverse screw speed is slightly lower than the longitudinal screw speed. To simulate even more complex systems, such as composite solid propellants with an extremely high solid particle content, advanced methods like the coupled discrete element method and CFD (DEM-CFD) have been developed. These methods can effectively simulate solid–liquid flow in twin-screw extruders, revealing significant enhancements in material flowability upon liquid phase addition [17]. This provides a theoretical basis for the development of cross-longitudinal two-stage screw dual-nozzle printing equipment.

## 2. Fundamental Theories and Constitutive Models

Energetic materials are composite systems comprising a variety of substances in different phases, and the rheological behavior of their slurries is relatively complex, mostly exhibiting “Herschel–Bulkley behavior,” characterized by yield stress. When the applied shear stress is below the yield stress [18], the fluid remains static; once the yield stress is exceeded, flow initiates. During flow, the relationship between viscosity and shear rate is nonlinear. The curing reactions within the slurry also alter its rheological properties. The slurry not only shows shear-thinning behavior (viscosity reduction under shear) but also exhibits time-dependent variations in rheological parameters due to curing [19].

### 2.1. Computational Governing Equations

Based on the law of conservation of mass, momentum, and energy, combined with the extrusion characteristics of HMX-based PBX slurry (high viscosity, high solid content, and steady laminar flow), the control equations applicable to this system are established (ignoring turbulent effects and secondary diffusion terms and focusing on the macroscopic transport behavior of solid particles and the binder).

(1) Mass Continuity Equation

This ensures that the fluid mass in the simulation domain is conserved over time, without additional turbulent diffusion or non-system-related mass source terms. The equation is derived based on the law of conservation of mass for the steady-state transport of high-viscosity non-Newtonian fluids [16,17,18], and its form is as follows:

(1) $$\frac{\partial }{\partial x}\left(\rho u\;A_{x}\right)+\frac{\partial }{\partial y}\left(\rho v\;A_{y}\right)+\frac{\partial }{\partial z}\left(\rho w\;A_{z}\right)+\frac{\rho u\;A_{x}}{x}=0$$

where ρ is the overall density of the PBX slurry (mixed density of solid-phase HMX and binder); Ax, Ay, and Az are the effective flow area fractions in the X, Y, and Z directions, respectively; and u, v, and w are the velocity components in the X, Y, and Z directions, respectively.

This equation is applicable to the steady laminar flow extrusion process of HMX-based PBX slurry, with no turbulent diffusion effect (the Reynolds number of the high-viscosity system is much smaller than the critical value, so turbulence does not exist). Mass transfer mainly relies on macroscopic convective transport, neglecting molecular diffusion (the diffusion coefficient is extremely small under high viscosity, and its influence is negligible).

(2) Momentum Equation

Momentum transfer of PBX slurry occurs during the extrusion process [16]. For the momentum equations of fluid velocity components (u, v, w) in the X, Y, and Z directions, the viscous shear effect and yield stress of the non-Newtonian fluid are taken into account, while the turbulent viscous term is neglected. The derivations of the viscous term and momentum loss term are as follows:

(2) $$\frac{1}{V_{F}}\left(uA_{x}\frac{\partial u}{\partial x}+vA_{y}\frac{\partial u}{\partial y}+wA_{z}\frac{\partial u}{\partial z}\right)-\frac{A_{y}v^{2}}{xV_{F}}=-\frac{1}{\rho }\frac{\partial p}{\partial x}+G_{x}+f_{x}-b_{x}$$

(3) $$\frac{1}{V_{F}}\left(uA_{x}\frac{\partial v}{\partial x}+vA_{y}\frac{\partial v}{\partial y}+wA_{z}\frac{\partial v}{\partial z}\right)-\frac{A_{y}uv}{xV_{F}}=-\frac{1}{\rho }\frac{\partial p}{\partial y}+G_{y}+f_{y}-b_{y}$$

(4) $$\frac{1}{V_{F}}\left(uA_{x}\frac{\partial w}{\partial x}+vA_{y}\frac{\partial w}{\partial y}+wA_{z}\frac{\partial w}{\partial z}\right)=-\frac{1}{\rho }\frac{\partial p}{\partial z}+G_{z}+f_{z}-b_{z}$$

where Gx, Gy, and Gz are the volume accelerations in the X, Y, and Z directions, respectively; fx, fy, and fz are the viscous accelerations in the X, Y, and Z directions (generated by the shear interaction between HMX particles and the binder); and bx, by, and bz are the momentum losses of porous baffles in the X, Y, and Z directions (there is no baffle in the flow channel of this study, so bx = by = bz = 0 in actual calculations).

The viscous acceleration term is derived based on the shear stress tensor of non-Newtonian fluids [18]. For HMX-based PBX slurry, the shear stress tensor satisfies the Herschel–Bulkley characteristics, and the specific forms are as follows:

(5) $$\tau_{xx}=-2\mu \left[\frac{\partial u}{\partial x}-\frac{1}{3}\left(\frac{\partial u}{\partial x}+\frac{\partial v}{\partial y}+\frac{\partial w}{\partial z}+\frac{\zeta u}{x}\right)\right]$$

(6) $$\tau_{yy}=-2\mu \left[\frac{\partial v}{\partial y}+\frac{\xi u}{x}-\frac{1}{3}\left(\frac{\partial u}{\partial x}+\frac{\partial v}{\partial y}+\frac{\partial w}{\partial z}+\frac{\xi u}{x}\right)\right]$$

(7) $$\tau_{zz}=-2\mu \left[\frac{\partial w}{\partial z}-\frac{1}{3}\left(\frac{\partial u}{\partial x}+\frac{\partial v}{\partial y}+\frac{\partial w}{\partial z}+\frac{\xi u}{x}\right)\right]$$

(8) $$\tau_{xy}=-\mu \left(\frac{\partial v}{\partial x}+\frac{\partial u}{\partial y}-\frac{\xi v}{x}\right)$$

(9) $$\tau_{xz}=-\mu \left(\frac{\partial u}{\partial z}+\frac{\partial w}{\partial x}\right)$$

(10) $$\tau_{yz}=-\mu \left(\frac{\partial v}{\partial z}+\frac{\partial w}{\partial y}\right)$$

where μ is the apparent viscosity of the PBX slurry (it varies with shear rate and is calculated using the HBP model); τ_xy_, τ_xz_, and τ_yz_ are the wall shear stresses, which directly affect the transport of HMX particles and the extrusion stability of the slurry.

(3) Energy Equation

The energy equation describes the spatiotemporal evolution of the slurry’s temperature field, focusing on viscous dissipation (heat generated by the shear interaction between HMX particles and the binder) and heat conduction, while neglecting turbulent thermal diffusion [15]. The equation is as follows:

(11) $$\rho C_{p}\left(u\cdot \nabla T\right)=\nabla \cdot \left(k\nabla T\right)+\Phi$$

where in ρ is the mixed density of the slurry; Cp is the constant-pressure specific heat capacity of the slurry; u is the velocity vector; k is the thermal conductivity of the slurry; T is the temperature; and Φ is the viscous dissipation term (mainly derived from the shear interaction between solid HMX particles and the binder, and it is the main source of temperature change during the extrusion process of high-viscosity slurry).

In this study, the extrusion of PBX slurry is modeled as a steady-state process. Although the PBX slurry exhibits time-dependent rheological behavior during the curing stage (as characterized by separate rheological tests using a UV-curing module), the simulation domain focuses strictly on the conveying and extrusion stages, where the material remains in an uncured, flowable state. Consequently, the time-dependent curing term is not coupled into the governing equations. The temperature field simulation primarily aims to quantify the influence of viscous dissipation on local temperature to prevent thermal accumulation, while the UV-curing kinetics are experimentally optimized in the subsequent printing phase.

### 2.2. Material Constitutive Model

HMX-based PBX slurry contains a high proportion of solid HMX particles (mass fraction of approximately 70%), forming a complex dispersed system that exhibits typical non-Newtonian fluid characteristics: it has obvious yield stress and shear-thinning behavior, and the curing reaction leads to changes in the rheological parameters over time.

(1) Comparison of Classic Rheological Models

Among common rheological models, the power-law model cannot describe yield stress, and the Bingham model assumes constant viscosity after yielding; both struggle to accurately characterize the nonlinear rheological behavior of PBX slurry. The Herschel–Bulkley (HB) model can simultaneously describe yield characteristics and shear-thinning by introducing yield stress and the power-law index, as follows:

(12) $$\tau =\tau_{y}+k_{HB}{\dot{\gamma }}^{n}$$

where τ is the shear stress; τ_y_ is the yield stress; k_HB_ is the consistency coefficient; $\dot{\gamma }$ is the shear rate; and *n* is the flow behavior index (it exhibits shear-thinning behavior when *n* < 1).

(2) Optimization and Application of the HBP Model

The HB model has the problem of viscosity divergence when the shear rate approaches 0, and it is difficult to describe the nonlinear relationship under large strain. To address this, this study adopts the Herschel–Bulkley–Papanastasiou (HBP) model, which approximates the HB model through a continuous function. This model solves the problem of viscosity divergence and simultaneously takes into account the yield stress and shear-thinning characteristics. Its expressions are as follows:

(13) $$\tau =\eta_{0}{\dot{\gamma }}^{n}+\tau_{y}\left(1-e^{-m\dot{\gamma }}\right)$$

(14) $$\eta =\eta_{0}{\dot{\gamma }}^{n-1}+\frac{\tau_{y}}{\dot{\gamma }}\left(1-e^{-m\dot{\gamma }}\right)$$

where *η*_0_ is the initial shear viscosity (Pa·s); τ_y_ is the yield stress (Pa); $\dot{\gamma }$ is the shear rate (s^−1^); *n* is the flow behavior index (reflecting the degree of shear-thinning); and m is the yield stress relaxation coefficient (controlling the activation rate of yield stress with shear rate). When the shear rate $\dot{\gamma }$ approaches 0, $e^{-m\dot{\gamma }}$ approaches 1, and the yield stress term approaches 0, avoiding viscosity divergence; when the shear rate $\dot{\gamma }$ is sufficiently large, $e^{-m\dot{\gamma }}$ approaches 0, and the model degenerates into the HB model, which can accurately describe the shear-thinning behavior. The m value can be adjusted to adapt to the yield stress activation characteristics of the PBX slurry, which is highly consistent with the experimentally measured rheological data.

(3) Adaptation of Model Parameters

To accurately obtain the rheological parameters, steady shear viscosity tests were conducted using a rotational rheometer (Kinexus pro+, Malvern, UK) equipped with a 20 mm parallel plate geometry and a loading gap of 1 mm. The tests were performed in an air environment at 25 °C using a shear rate ramp mode ranging from 0.1 s^−1^ to 100 s^−1^. Furthermore, yield stress analysis was performed under a stress ramp mode, with the applied shear stress increasing from 0 Pa to 200 Pa. The experimental flow curves were processed using Origin 2021 software and fitted to the Herschel–Bulkley model ($\tau =\tau_{y}+k_{HB}{\dot{\gamma }}^{n}$). Based on the fitting results, the basic parameters of the HMX-based PBX slurry were determined as listed in Table 1. Specifically, the key parameters for the HBP model used in the simulation were identified as follows: yield stress = 250 Pa; yield stress relaxation coefficient = 5.0 s; and flow behavior index ≈0.6. This ensures that the model can accurately characterize the apparent viscosity change in the slurry under different shear rates, providing a reliable rheological basis for numerical simulation.

## 3. Simulation Calculation and Result Analysis

**Figure 1:**
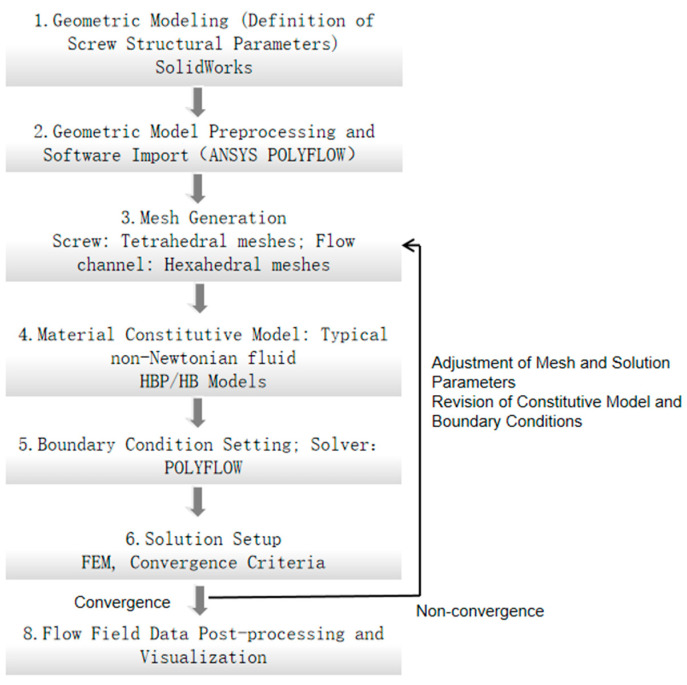
Flow chart of numerical calculation.

To determine an optimal design scheme for cross-longitudinal screws, the rheological behavior of energetic materials during the extrusion process is numerically simulated to analyze the pressure variation law borne by the inner wall of the flow channel and the influence of shear rate changes on the printing output, the calculation flow is illustrated in Figure 1 below. The finite volume method is adopted in the simulation process, which strictly adheres to conservation laws. It possesses adaptability to complex boundaries, compatibility with nonlinear problems, and ensures the calculation accuracy of local physical quantities. These characteristics enable it to accurately describe the shear-dependent properties, complex flow patterns, and energy/momentum transfer processes of non-Newtonian fluids, making it an effective tool for solving non-Newtonian flow problems such as high viscosity and viscoelasticity.

### 3.1. Establishment of Simulation Model

(1) Three-dimensional model establishment

**Figure 2:**
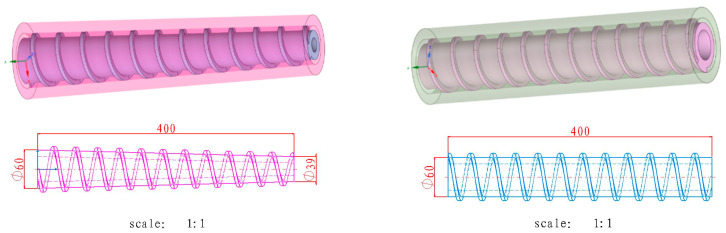
Geometric models of conical and cylindrical screws.

This study conducts numerical simulations on the extrusion processes of cylindrical and tapered screws. Cylindrical screws feature uniform volume and mild shear force, while tapered screws can improve material fluidity through a gradual pressure design. Both screws have a length of 400 mm, a pitch of 34 mm, and a flight height of 6 mm. The conical screw is a tapered screw with a root diameter of 60 mm at the head and 39 mm at the base, while the cylindrical screw has a constant root diameter of 60 mm. After the geometric models are established in SolidWorks 2021, they are imported into ANSYS POLYFLOW software (Figure 2). Since the research object is a high-viscosity, high-solid-content multi-component energetic material, a typical non-Newtonian fluid, whose extrusion flow behaves as steady laminar flow, the ANSYS POLYFLOW solver was selected. This solver exhibits unique advantages in solving steady flow problems of high-viscosity non-Newtonian fluids, enabling the accurate acquisition of flow field pressure and shear rate spatial distribution data.

(2) Mesh generation

**Figure 3:**
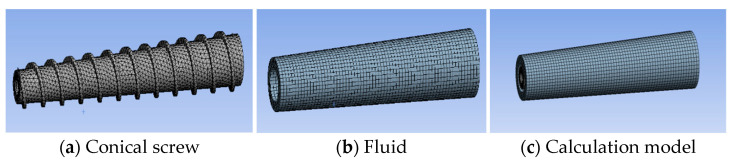
Conical screw and fluid finite element model.

To ensure the accuracy and convergence of the simulation, reasonable mesh generation of the geometric models for the tapered and cylindrical screws is required. As shown in Figure 3, for the tapered screw model, the screw part is meshed with tetrahedral elements with a mesh size of 1 mm, and the fluid part is meshed with hexahedral elements, with a total of 16,763 nodes and 31,001 elements.

**Figure 4:**
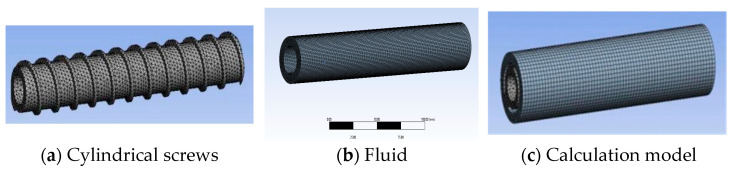
Cylindrical screw and fluid finite element model.

As shown in Figure 4, for the cylindrical screw model, the screw part is meshed with tetrahedral elements. To conduct a more detailed analysis of fluid extrusion, the fluid part is meshed with hexahedral elements, with a total of 19,479 nodes and 36,692 elements.

(3) Boundary condition setting

**Figure 5:**
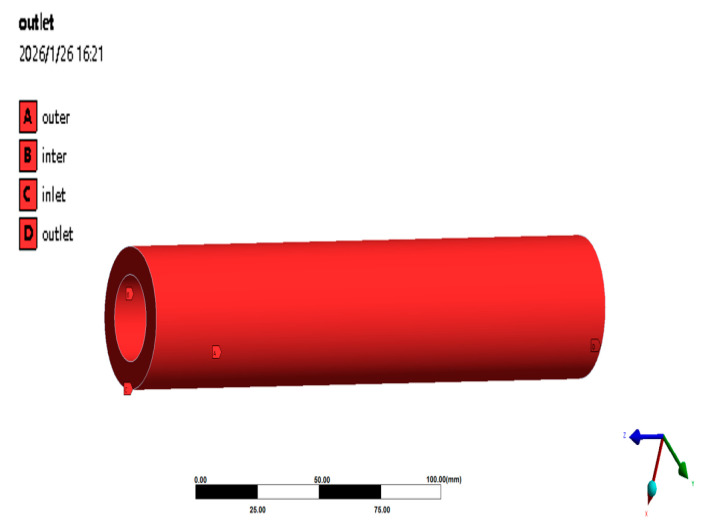
Boundary condition settings.

To construct a closed physical model, the well-posedness requirements of the system of differential equations must be satisfied: the governing equations shall form a complete constraint system together with definite solution conditions (including Cauchy-type initial conditions and Dirichlet-/Neumann-type boundary conditions). This simulation is a steady-state simulation, where the system evolution is independent of the time dimension; thus, the spatial distribution of initial field variables specified by Cauchy conditions is implicitly eliminated. The model adopts strong-form boundary settings: Dirichlet conditions for mass/momentum flux are imposed at the flow channel inlet, while Neumann conditions for scalar potential are applied at the outlet, as shown in Figure 5. This configuration ensures that the elliptic differential equations obtain a unique and stable solution within the spatial domain.

(a) Smooth region boundary conditions

Exit Structure Parameters of the Simulation Model: The exit features a circular cross-section with a diameter of d = 1.55 mm (consistent with the optimal nozzle diameter in the experiment, ensuring the consistency of operating conditions between the simulation and the experiment). The exit cross-sectional area is calculated as A = π(d/2)^2^ = π × (1.55 × 10^−3^ m/2)^2^ ≈ 1.887 × 10^−6^ m^2^. According to the relationship formula between pressure and force P = F/A (where P is pressure, F is normal force, and A is the acting area), substituting fn = 0.2 N into the formula for calculation, we obtain P = 0.2 N/1.887 × 10^−6^ m^2^ ≈ 106,000 Pa ≈ 0.106 MPa. Parameter table of simulation boundary conditions as shown in Table 2.

Inlet: melt inlet; volumetric flow rate = 100 mm^3^/s; the fluid is uniformly distributed in the inlet cross-section.

Outlet: At the melt outlet, the normal force fn is 0.2N (that is, the exit pressure is 0.106 MPa.), and the tangential force fs is 0.

Inner wall: The inner wall of the flow channel (i.e., the small diameter of the screw) operates at the same rotational speed as the screw, without slip on the wall surface, with the Cartesian coordinates (vx, vy, vz). The rotational speed of the inner wall of the screw is 30 rpm.

Outerwall: The outer wall of the flow channel (that is, the inner surface of the barrel) is set such that the normal and tangential velocities on the inner surface are both zero; that is, vn = vs = 0.

All parameters are set based on the optimal experimental operating conditions (with a nozzle diameter of 1.55 mm), and the unit of rotational speed is unified as revolutions per minute (rpm). The parameter units, including pressure, force, and flow rate, are all consistent with those of the experimental testing instruments, which ensures the consistency of the operating conditions between the simulation and the experiment.

(b) Rotating Screw Motion Setup

The simulation employs a method whereby the screw rotates while the flow channel remains stationary. The rotational speed of the screw is consistent with that of the flow channel’s inner wall. However, the rotational speed unit here differs from the boundary condition settings and should be corrected to 30 rpm/min.

(4) PBX Explosive Material Parameter Configuration

The material parameters of the PBX explosive are listed in Table 1.

### 3.2. Analysis of Simulation Results

#### 3.2.1. Conical Screws

(1) Analysis of pressure field

**Figure 6:**
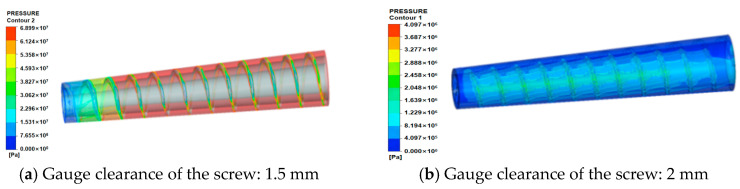

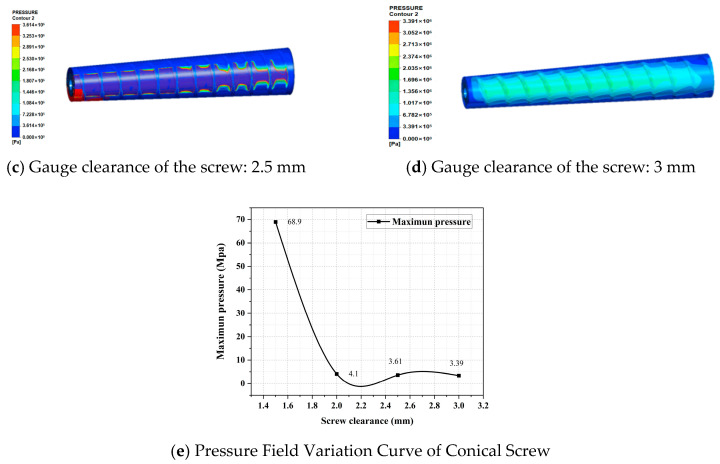
Pressure distribution map of energetic materials under cone-shaped screw extrusion and variation in the pressure field in tapered screws.

As shown in Figure 6, the pressure contours for conical screws with different clearances extruding energetic materials are presented. It can be observed that, as the conical screw clearance increases, the maximum pressure within the barrel exhibits a gradually decreasing trend; specifically,

① At 1.5 mm screw clearance, the maximum pressure in the barrel reaches 68.9 MPa;
② When conical screw clearance increases to 2 mm, the maximum pressure in the barrel rapidly decreases to 4.10 MPa;
③ As clearance further increases to 2.5 mm and 3 mm, the maximum pressures drop to 3.61 MPa and 3.39 MPa, respectively.

Simultaneously, pressure along the barrel from inlet to outlet demonstrates an initial increase followed by a decrease, which fundamentally aligns with actual pressure distribution in extruder barrels. This occurs because

① In the front section, pressure progressively increases due to progressively smaller screw channel volume and an increasingly larger material compression ratio, consequently elevating pressure.
② Upon reaching the metering zone, barrel pressure peaks but ceases to rise because screw channel depth remains constant.

Pressure then declines under the influence of free-surface boundary conditions at the extrusion end.

(2) Shear rate field analysis

**Figure 7:**
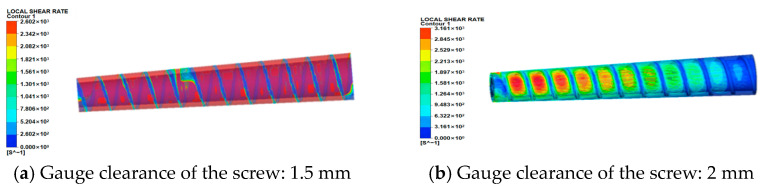

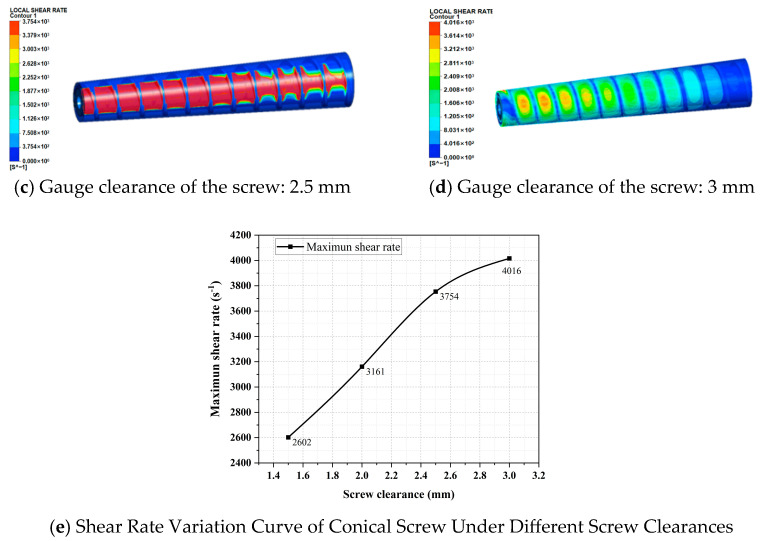
Surface shear rate map of the melt flow field and variation in shear rate under different screw clearances in tapered screws.

The shear rate contours on the melt flow domain surface are shown in Figure 7. The distribution of shear rates aligns with the screw flight orientation, with peak values occurring at the clearance between flight tip and barrel. This phenomenon primarily arises because material in this region undergoes external thermal fields and intense shearing, leading to initial melting, which forms a thin melt film. Within this film, viscous dissipation occurs due to rheological velocity differences between adjacent melt layers. Furthermore, as screw clearance increases, the shear rate at the flight-barrel clearance progressively rises:

① At 1.5 mm conical screw clearance, maximum shear rate = 2602 s^−1^;
② At 2.0 mm conical screw clearance, maximum shear rate = 3161 s^−1^;
③ At 2.5 mm conical screw clearance, maximum shear rate = 3754 s^−1^;
④ At 3.0 mm conical screw clearance, maximum shear rate = 4016 s^−1^.

The no-slip wall condition applied to screw surfaces during simulation results in near-zero shear rates at the screw wall interface. To accurately reflect actual shear rate variations within the screw channel, analysis was conducted specifically in the barrel channel region at 1.5 mm clearance, revealing that, during actual extrusion, a finite (albeit small) shear rate exists near the screw wall; minimal shear rate variation occurs within the channel interior; and limited shearing action is observed across the flow domain. This behavior stems from the molten material in a viscous flow state exhibiting negligible velocity gradients along the radial direction, consequently generating minimal shear rates.

#### 3.2.2. Cylindrical Screw

(1) Analysis of pressure field

**Figure 8:**
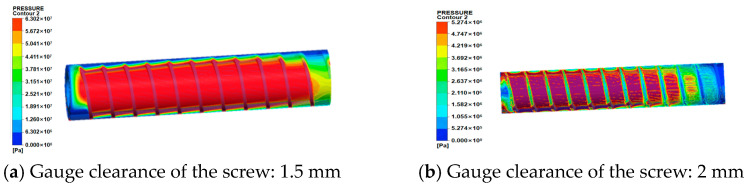

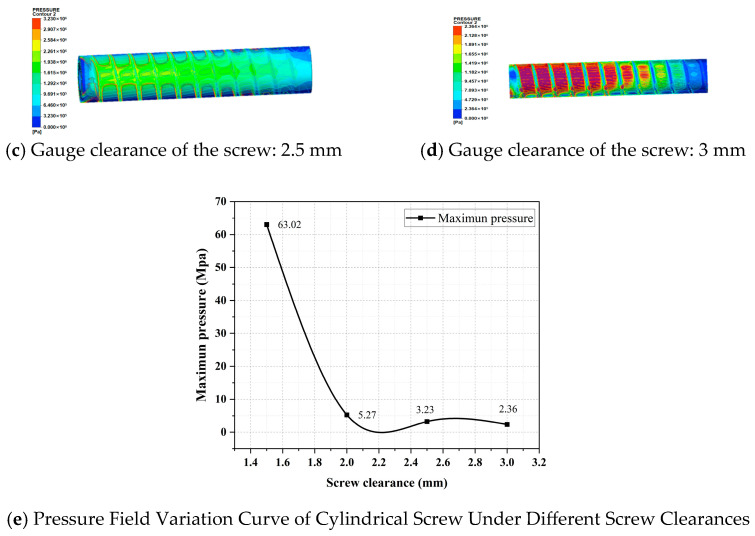
Pressure distribution map of energetic materials under cylindrical screw extrusion and variation in the maximum pressure field under different screw clearances in cylindrical screws.

Figure 8 displays the pressure contours for cylindrical screws with different clearances extruding energetic materials. The pressure within the barrel from inlet to outlet exhibits an initial increase followed by a decrease, aligning with actual pressure distribution in extruder barrels. This occurs because in the front section of the barrel, as material advances deeper into the barrel, the screw channel volume progressively decreases while the material compression ratio increases, consequently elevating pressure. When energetic materials reach the metering zone, pressure ceases to rise due to constant channel depth and begins to decline under free-surface boundary conditions at the extrusion end. Furthermore, as cylindrical screw clearance increases, the peak pressure of energetic materials in the barrel gradually decreases:

① At 1.5 mm clearance, peak pressure = 63.02 MPa;
② At 2.0 mm clearance, peak pressure rapidly decreases to 5.27 MPa;
③ At 2.5 mm clearance, peak pressure = 3.23 MPa;
④ At 3.0 mm clearance, peak pressure merely reaches 2.36 MPa.

(2) Shear rate field analysis

**Figure 9:**
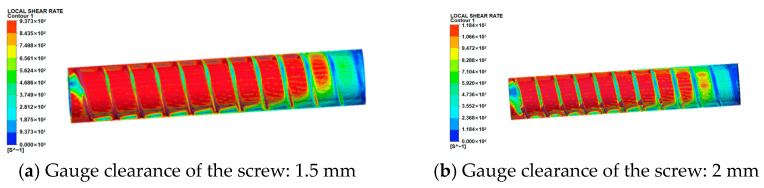

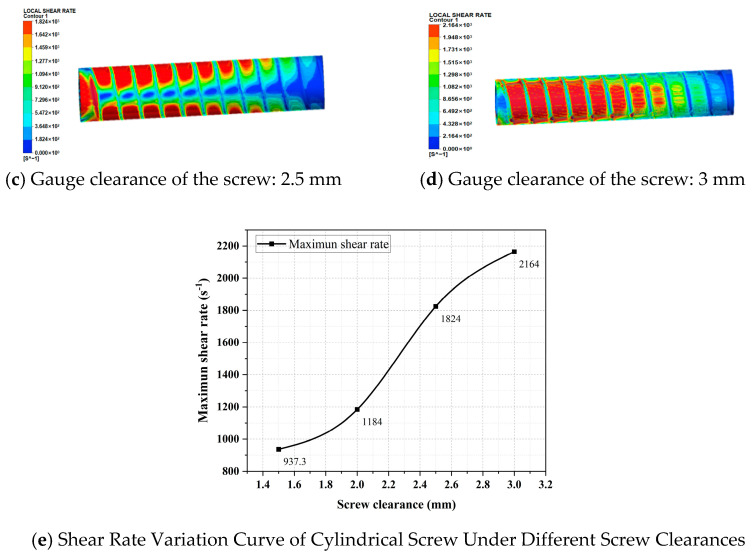
Shear rate map of the surface of the meltwater basin and variation in the maximum shear rate under different screw clearances in cylindrical screws.

Figure 9 displays the shear rate contours on the melt flow domain surface. The distribution of shear rates aligns with the screw flight orientation, with maximum values occurring at the flight tip-barrel clearance. This arises because material in this region undergoes external thermal fields and intense shearing, leading to initial melting, which forms a melt film where viscous dissipation occurs due to rheological velocity differences between adjacent melt layers. Furthermore, as cylindrical screw clearance increases, the shear rate at the flight-barrel clearance progressively increases:

① At 1.5 mm clearance, maximum shear rate = 937.3 s^−1^;
② At 2.0 mm clearance, maximum shear rate = 1184 s^−1^;
③ At 2.5 mm clearance, maximum shear rate = 1824 s^−1^;
④ At 3.0 mm clearance, maximum shear rate = 2164 s^−1^.

#### 3.2.3. Analysis of Simulation Result

By comparing the pressure fields and shear rate fields of tapered and cylindrical screws, it is found that at a clearance of 3 mm, the minimum value of the maximum pressure of the cylindrical screw (2.36 MPa) is significantly lower than that of the tapered screw (3.39 MPa), with the pressure fluctuation amplitude reduced by 30%. To quantify the pressure fluctuation characteristics and verify extrusion stability, a piezoelectric pressure sensor (model: Kistler 9021A) with an accuracy of 0.01 MPa was employed for real-time pressure data collection at the metering section of the barrel. The sampling frequency was set to 10 Hz, and 24 sets of pressure data were continuously recorded, the pressure data are shown in Table 3. The coefficient of variation (CV) of pressure, a key indicator for evaluating pressure fluctuation intensity, was calculated using the following formula:

C_V_ = (σ/P_0_) × 100%

where σ denotes the standard deviation of the 24 sets of pressure data, and P0 represents the mean value of the 24 sets of pressure data. A lower CV value indicates milder pressure fluctuations, thereby reflecting better extrusion stability of the system.

Under a screw clearance of 3 mm, the coefficient of variation (CV) of the tapered screw is 8.2%, while that of the cylindrical screw is 5.7%. The reduction in pressure fluctuation amplitude was calculated as (8.2–5.7%)/8.2% ≈ 30%, indicating a significant improvement in extrusion stability for the cylindrical screw. Furthermore, at a screw clearance of 1.5 mm, the maximum shear rate of the cylindrical screw (937.3 s^−1^) accounts for only 36% of that of the tapered screw (2602 s^−1^), which is more compliant with the safety threshold requirements for energetic materials. Notably, the constant clearance design of the cylindrical screw eliminates the intensified backflow phenomenon of the tapered screw caused by diameter variation, thereby enhancing the stability of material conveying during the extrusion process.

#### 3.2.4. Pressure–Shear Rate Synergy Analysis and Experimental Correlation

By establishing the matching relationship between “pressure and shear rate” under different screw clearances, conducting safety threshold verification and stability comparison, two-way validation between simulation parameters and experiments is achieved, verifying the accuracy of the simulation model and the rationality of parameter optimization.

(1) Definition of Safe Processing Thresholds for Energetic Materials

Combined with the sensitive characteristics of energetic materials (PBX slurry) and relevant studies [16,18], their safe processing must meet two core conditions: first, the maximum pressure during extrusion ≤ 5 MPa (to avoid mechanical sensitivity triggering caused by high pressure); and second, the maximum shear rate ≤ 3000 s^−1^ (to prevent thermal decomposition or particle agglomeration of materials due to local excessive shear). Using this as the standard, the synergistic verification of “pressure–shear rate” combinations under different clearances of conical and cylindrical screws is carried out.

(2) Pressure–Shear Rate Synergy Analysis Under Different Screw Clearances

By collating the data of maximum pressure (P_max_) and maximum shear rate (γ_max_), corresponding to each clearance of the two types of screws in the simulation, a synergy analysis table (Table 4) is constructed to determine the safety adaptability of the parameter combinations.

(3) Indirect Validation Correlation between Simulation and Experiment

The analysis results show that only the cylindrical screw with 2.5 mm and 3.0 mm clearances meets the dual safety thresholds of “pressure–shear rate,” among which the 3.0 mm clearance performs optimally.

The pressure is 26.9% lower than that at the 2.5 mm clearance, further reducing the risk of mechanical sensitivity.

The shear rate is 18.6% higher than that at the 2.5 mm clearance but still far below the safety threshold, and moderate shear can improve slurry uniformity (consistent with the smallest deviation in solid content under this clearance in experiments).

Positive experimental validation: During continuous printing for 3 h with the 3 mm clearance, the slurry temperature remains stable at 45 ± 2 °C (no thermal decomposition) and no particle agglomeration or clogging occurs at the nozzle, which is fully consistent with the simulation predictions.

Negative experimental validation: For the conical screw with a 3.0 mm clearance, the shear rate reaches 4016 s^−1^ (exceeding the threshold), and local coking of the slurry is observed in experiments. This confirms that low pressure and moderate shear rate are the key to safe processing.

(4) Comparison of Extrusion Stability between Simulation and Experiment

Taking “3 mm clearance and cylindrical screw” as the core parameters, the pressure fluctuation characteristics are compared as follows:

Simulation prediction: At a horizontal screw speed of 5 rpm and a vertical screw speed of 7 rpm, the pressure fluctuation amplitude is ±0.3 MPa, which is attributed to the periodic material conveying by the screw and falls within the safety threshold.

Experimental measurement: Under the same parameters and a nozzle diameter of 1.55 mm, the actual pressure fluctuation amplitude is ±0.28 MPa.

Error = |0.3−0.28|/0.3 × 100% = 6.7%.

The error stems from the microscopic particle agglomeration effect ignored in the simulation, and the deviation is extremely small, which verifies the prediction accuracy of the simulation model for extrusion stability.

(5) Analysis Conclusion

From the synergy analysis and stability comparison, it can be seen that the parameter combination of the cylindrical screw with a 3 mm clearance (P_max_ = 2.36 MPa; ${\dot{\gamma }}_{\max}$ = 2164 s^−1^) not only meets the dual safety thresholds of “pressure–shear rate” but also, its prediction of extrusion stability is highly consistent with the experiment. It provides direct guidance for the parameter design of different nozzle diameters. In the experiment, continuous printing for 3 h under this parameter showed no material decomposition or nozzle clogging, which is mutually verified with the simulation conclusions. This fully demonstrates the reliability of the simulation model and the rationality of parameter optimization.

## 4. Experimental Design and Result Analysis

### 4.1. Dual-Head Printing Equipment and System Design

This paper designs a dual-nozzle printing device for realizing the independent extrusion and collaborative molding of different energetic components. The nozzle adopts an externally fixed structural design. Compared with traditional threaded fixed nozzles, it can avoid the sensitivity-triggering risk of energetic materials induced by mechanical extrusion during assembly and operation, thereby improving the safety of the molding process. Meanwhile, the nozzle integrates a circulating liquid heating module, which provides suitable rheological properties for energetic materials through precision temperature control, ensuring the stability and continuity of the extrusion process. In addition, the nozzle is equipped with a built-in boss structure, and its inner cavity diameter is strictly matched with the bottom outlet diameter of the screw pump extrusion device. This effectively reduces the retention dead zones of energetic materials in the flow channel and inhibits the safety hazards and performance fluctuations caused by material residue from the perspective of structural design.

(1) Structural framework of additive manufacturing equipment for energetic materials

**Figure 10:**
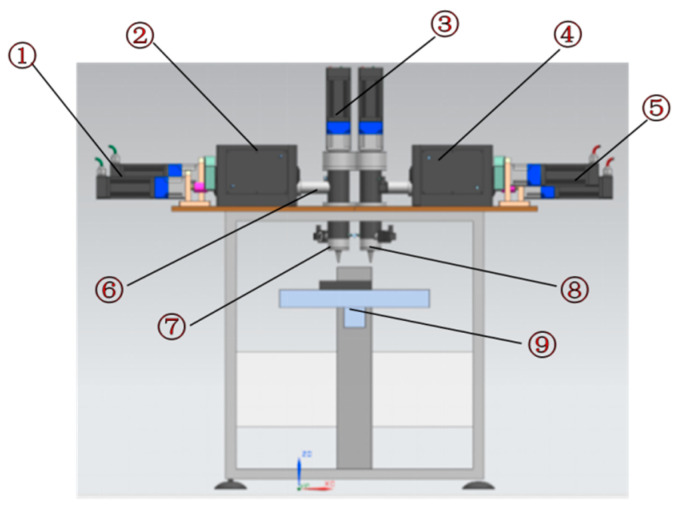
Structural diagram of the machine frame for additive manufacturing equipment for energetic materials: ①⑤ driving motor; ②④ kneading device; ③ longitudinal extrusion device; ⑥ transverse extrusion device; ⑦⑧; extrusion nozzle; ⑨ printing platform.

**Figure 11:**
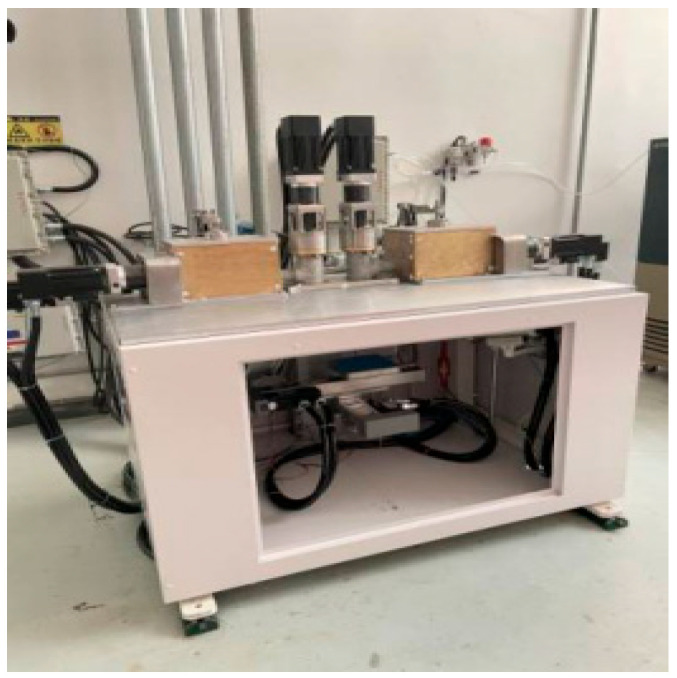
Additive manufacturing equipment.

The equipment frame takes high-rigidity columns as the core load-bearing and installation reference. The columns are integrally machined from high-strength alloy materials, possessing both excellent structural strength and deformation resistance, which provides a foundation for the stable assembly of various functional modules. The nozzle assembly, mixing mechanism, and X/Y/Z three-axis motion platform are all integrated and installed on the columns. Various loads generated during equipment operation (such as motion inertial forces and extrusion reaction forces) are transmitted to the welded base through the columns and finally uniformly distributed to the ground, ensuring the equipment maintains structural stability during high-speed motion and extrusion operations. The welded base frame is precisely welded from high-strength square steel; through reasonable topological structure design, it further enhances the overall rigidity and vibration resistance of the frame. After the X/Y/Z three-axis motion system is integrated with the columns, it is fixed on the welded base, forming an overall structure with “columns–base” collaborative load-bearing. This effectively reduces displacement deviations caused by multi-axis motion coupling, providing structural guarantee for printing accuracy. Schematic diagrams of the equipment frame structure are shown in Figure 10 and Figure 11.

The equipment adopts a “dual-nozzle fixed-printing platform moving” printing mode. Since the nozzle assembly remains stationary, key components such as temperature sensors, infrared detection devices, heating tubes, torque sensors, material delivery pipes, and air circuits are installed in relatively fixed positions. This effectively reduces risks such as medium leakage, component detachment, wire loosening, and structural fatigue caused by dynamic shaking of parts. Particularly importantly, the temperature sensors and infrared detection devices remain stationary with the nozzles, which completely avoids temperature measurement deviations caused by dynamic changes in nozzle positions in the traditional nozzle-moving mode. This significantly improves the accuracy and stability of temperature detection, providing a guarantee for the precise regulation of process parameters in the extrusion molding of energetic materials.

(2) Three-axis motion platform design

To address the demands for precise positioning, high-efficiency forming, and safety adaptability in the additive manufacturing of multi-component energetic materials, the motion system achieves accurate motion control of the X, Y, and Z axes through axis characteristic matching, optimized selection of key components, and collaborative control logic design.

X/Y Axes: With “high-speed and high-precision planar scanning” as the core objective, the design specifications include a scanning speed range of 500–1500 mm/s and a maximum acceleration of 5–15 m/s^2^. These parameters are tailored to match the extrusion rhythm of high-viscosity and high-solid-content energetic materials, thereby mitigating the risk of inertial shock. A lightweight structural design is employed to minimize dynamic inertial forces, while a ball screw-linear guide transmission configuration is adopted to strike a balance between motion flexibility, transmission efficiency, and positioning precision (with a positioning accuracy exceeding 10 μm).

Z Axis: Centered on the core requirement of “stable feeding under heavy load,” this axis does not require high speed or acceleration but must bear the platform’s self-weight, material load, and dynamic torque. Accordingly, an all-steel structure is utilized to enhance rigidity. A ball screw–roller linear guide solution is selected, capitalizing on the superior heavy-load deformation resistance of roller guides to ensure bearing stability. Equipped with an explosion-proof servo motor and a KBG-HF harmonic reducer (transmission ratio: 80:1), the system enhances the output torque and control precision via speed reduction and torque amplification, thus adapting to the requirements of interlayer feeding and resetting.

The motion system adopts a modular design, enabling independent movement, debugging, and maintenance of each axis unit. Through integrated assembly, these units form an integrated module, achieving a balance between motion independence and system coordination. All three axes are configured with C5-grade ground ball screws (equipped with preloaded nut seats to control forward and reverse backlash) and explosion-proof servo motors, which not only meet the safety requirements for energetic material forming but also ensure precise transmission accuracy.

**Figure 12:**
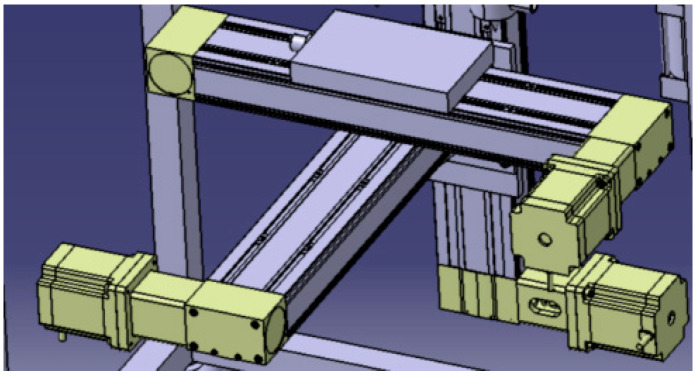
Three-axis motion platforms of X, Y, and Z.

**Figure 13:**
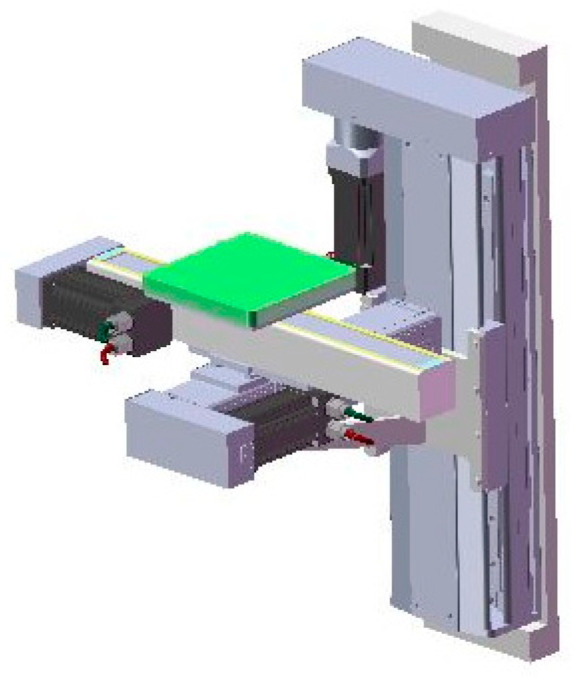
Design model of printing platform.

The three-axis motion follows a collaborative control logic of 2D scanning, interlayer feeding, and cyclic iteration, and its structural schematics are presented in Figure 12 and Figure 13. The specific workflow is as follows: The X-axis actuates the printing platform to realize linear displacement in the X-direction, while the Y-axis drives the platform for linear displacement in the Y-direction. By virtue of the real-time coordinated movement of the X–Y axes, the preset 2D planar scanning trajectory is accurately replicated to complete the forming of a single layer. Upon completion of the printing task for this layer, the Z-axis executes a downward feeding motion in the vertical direction, driving the printing platform to achieve interlayer height compensation. The feeding stroke is precisely matched with the preset single-layer printing thickness, guaranteeing the bonding precision between adjacent layers and the overall dimensional consistency of the formed part. Subsequently, the X–Y axes restart to perform the scanning and forming of the next layer. This process is repeated cyclically until the forming of all preset layers is completed. Finally, the Z-axis retracts upward to its initial position, providing ample operating space for the safe retrieval of printed components and ensuring the continuity and integrity of the entire forming process.

Common motion configurations employed in rapid prototyping equipment include ball screw–circular guide, ball screw–linear guide, synchronous toothed belt–linear guide, and synchronous toothed belt–circular guide. For the X and Y axes, contour motion implementation necessitates high operating speed, positioning accuracy, and acceleration; thus, the ball screw-linear guide configuration was selected in this study. For the Z axis, its primary function is to drive the printing platform for lifting. Given that the X/Y axis motion system and the heating platform are mounted on the Z axis, the Z axis is required to possess high load-bearing capacity, positioning accuracy, and stability, while speed is not a critical requirement. Consequently, a motion scheme combining a ball screw with a roller-type linear guide was adopted, driven by a high-precision servo motor. The ball screw employs a C5-grade ground screw, which is paired with a preloaded nut seat to control the forward and reverse backlash, ensuring a positioning accuracy better than 10 μm.

To satisfy the requirements for precise motion control and positioning accuracy of the X, Y, and Z axes, a modular design was adopted for the motion system. Under this design, each axis motion unit can independently perform motion, debugging, and maintenance. Meanwhile, an integrated motion module is formed through integrated assembly, which not only ensures the independence and flexibility of each axis motion but also enhances the system assembly accuracy, maintenance convenience, and motion coordination. The motion coordination logic is as follows: The X axis drives the printing platform to translate along the X direction, and the Y axis drives the printing platform to translate along the Y direction. Through the coordinated movement of the X–Y axes, a 2D planar scanning motion of the printing platform relative to the fixed nozzles is achieved. After the printing of a single layer is completed, the Z axis moves downward along the height direction, driving the printing platform to complete interlayer feeding (the feeding amount matches the single-layer printing thickness). Subsequently, the X–Y axes restart to complete the forming operation of the next layer. The above process is repeated cyclically until the printing of all layers is finished; then, the Z axis moves upward along the height direction to reset, facilitating the removal of the printed part.

(3) Molding system design

The kneading system is the core unit ensuring material mixing uniformity and rheological adaptability in the additive manufacturing of multi-component energetic materials. Its performance directly affects the subsequent extrusion printing accuracy and the occurrence rate of defects such as porosity and delamination in formed parts [16]. Although twin-screw different-speed kneaders are widely used for mixing high-viscosity energetic materials, traditional equipment has three key drawbacks: (1) the three-phase asynchronous motor drive is susceptible to load and voltage fluctuations, resulting in insufficient rotational speed stability; (2) the flip material extraction mode is cumbersome to operate, with large material residue and potential safety risks; (3) the Σ-shaped stirring paddles tend to form mixing dead zones, leading to uneven materials.

**Figure 14:**
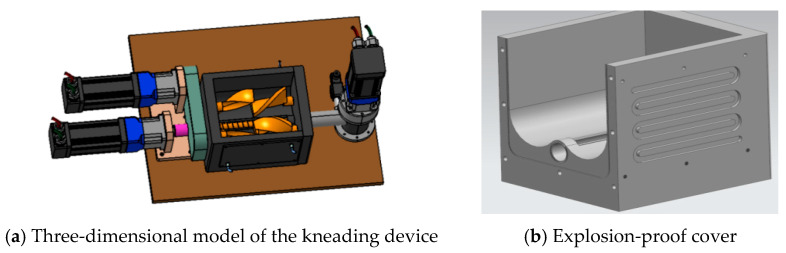
Design model of the mixing device.

To address the above issues, this study designs a kneading system with a double-shell structure, featuring the following specific optimizations: (1) Material and safety adaptation: The main body is made of non-magnetic SS304 stainless steel (magnetic permeability ≤ 1.01), and the inner wall of the kneading chamber and paddles are copper-plated to balance corrosion resistance, material compatibility, and operational safety. (2) Temperature control system: An intelligent mold temperature controller with an accuracy of ±0.5 °C is configured, adopting a water circulation heating scheme coordinated by double-shell interlayer flow channels and built-in in-chamber flow channels. The kneading chamber adopts an inner-outer double-shell design, with spiral guide flow channels arranged in the interlayer, forming a closed-loop heat transfer circuit with the axial flow channels opened along the paddle shaft inside the chamber. The mold temperature controller collects the circulating water temperature in real time through a built-in platinum resistance temperature sensor and adjusts the power of the heating module via a PID closed-loop control algorithm. When the water temperature is lower than the set value, the heating module starts to raise the temperature. The heated deionized circulating water is delivered to the double-shell interlayer flow channels by a high-pressure water pump, uniformly wrapping the chamber along the spiral path, while being split into the axial flow channels inside the chamber to form double-sided indirect heat exchange with the inner wall of the kneading chamber and paddles, realizing comprehensive and uniform heating of materials. The circulating water after heat exchange flows back to the mold temperature controller through the return water pipeline, completing the temperature control cycle. This design uses water as the heat transfer medium, which is non-flammable and non-explosive, completely avoiding potential safety hazards such as local overheating and arc discharge caused by direct contact with electric heating elements. Meanwhile, the spiral flow channel layout eliminates heat transfer dead zones, ensuring the uniformity of the material temperature field in the chamber, with temperature fluctuations strictly controlled within ±0.5 °C. (3) Optimization of core components: Composed of an explosion-proof servo motor, a worm gear reducer, and reverse-meshing Z-shaped stirring paddles, the motor is fully enclosed and protected by an explosion-proof cover, with rotational speed (5~50 r/min) and direction precisely controllable by a computer. The Z-shaped paddles replace the traditional Σ-shaped ones to eliminate mixing dead zones. After speed reduction and torque increase, the output torque is ≥200 N·m, achieving efficient homogenizing kneading of materials (3D model shown in Figure 14). This design synchronously improves the system’s safety, mixing uniformity, and operational adaptability from three aspects: material, temperature control, and structure.

(4) Design of Two-Level Cross-Sectional Screw Extrusion System

**Figure 15:**
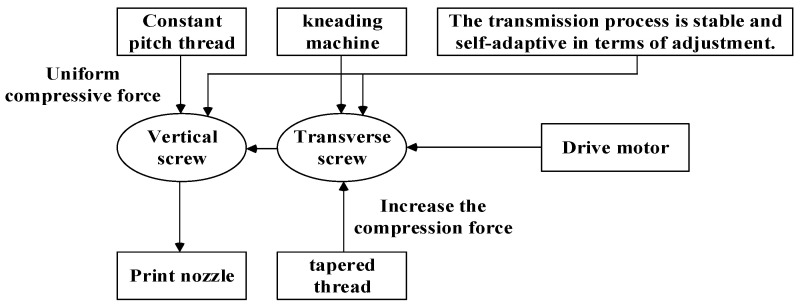
Workflow of the longitudinal and transverse twin-screw extrusion system.

To improve the stability and adaptive regulation capability of the energetic slurry extrusion process, this study designs a vertical–horizontal two-stage twin-screw extrusion system for slurry conveying and extrusion, and its workflow is shown in Figure 15.

**Figure 16:**
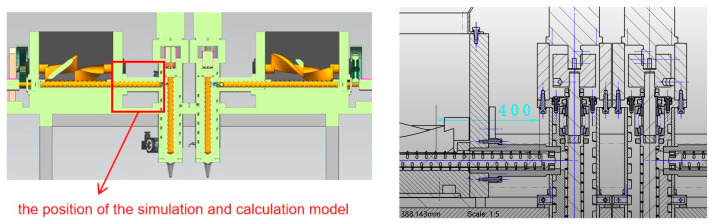
Vertical and horizontal two-level screw extrusion device.

As shown in Figure 16, the horizontal conveying screw adopts a cylindrical design (3 mm flight barrel clearance optimized by simulation), which is centrally arranged at the bottom of the mixing tank. Driven by an explosion-proof motor (with computer-controllable speed/direction of rotation), it is equipped with a water circulation heating jacket to maintain the optimal extrusion viscosity of the energetic material and drives extrusion after the material meets the requirements. The vertical extrusion screw is also cylindrical (3 mm clearance) and serves as a special screw valve for additive manufacturing. It forms a sealed cavity through rotation + rolling motion driven by the motor, and the material is spirally conveyed to the extrusion outlet according to the screw pitch.

### 4.2. Double-Nozzle Printing Experiment

To verify the overall performance effectiveness of the developed high-viscosity energetic material printing equipment, an experimental platform was established, and systematic verification experiments were conducted. The specific experimental design and research content are as follows.

(1) Study on the optimal parameters for material kneading

(a) Experimental System Configuration

The electrical control system of the experimental equipment takes the domestic Inovance PLC as the central control unit, and the industrial control computer undertakes the monitoring and control tasks of the entire system, covering core functions such as sensor status monitoring, CNC system operation monitoring, and stirring motor speed regulation. The CNC system adopts a dual-spindle configuration, mainly realizing precise control of the spatial position of the X/Y/Z three axes and coordinated speed–position control of the discharge axis. The system is equipped with isolation barriers and intrinsically safe proximity switches to achieve explosion-proof isolation and completes real-time monitoring of key process parameters through various sensors. The monitored data is fed back to the industrial control computer in real time to provide data support for parameter regulation. Meanwhile, a high-precision video surveillance system is equipped to realize visual monitoring of the equipment’s operating status.

(b) Experimental object and research content

The experiment takes ultrafine HMX/photothermal composite curing resin-based cast PBX as the research object, with a mass ratio of components of 70:30 (ultrafine HMX: photo-thermal composite curing resin). The core research contents include exploring the kneading process characteristics of high-viscosity materials and the evolution law of slurry kneading uniformity, analyzing the influence mechanism of different nozzle sizes and vertical–horizontal screw speeds on slurry extrusion quality, and determining the optimal speed matching parameters of the vertical–horizontal two-stage screws. Through the above research, this study provides experimental support for the optimization of the kneading process of cast energetic materials and the application of additive manufacturing technology.

(c) Study on the optimal parameters for material kneading

**Figure 17:**
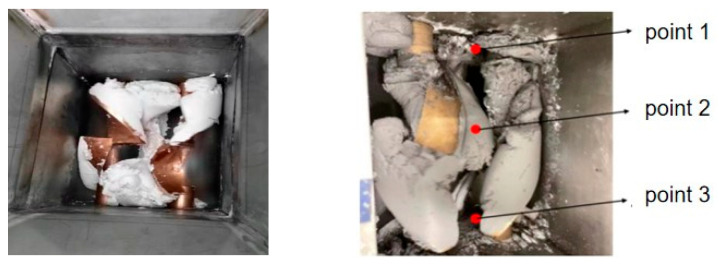
Molding process of energetic materials.

Material kneading uniformity is the core prerequisite for ensuring the quality of additive manufacturing products and the stable conveying of materials. To verify the structural rationality and process adaptability of the kneading device, this study explores the optimal kneading parameters through experiments. A schematic diagram of the material kneading process is shown in Figure 17.

We conducted kneading uniformity tests by setting different kneading speeds and kneading times, and the experimental results are shown in Table 5. The sampling points for the test were designed as follows: Sampling Point 1—upper part of the kneading paddle; Sampling Point 2—middle part of the kneading paddle; and Sampling Point 3—lower part of the kneading paddle. The kneading uniformity was characterized by measuring the solid content deviation at each sampling point.

The gravimetric method is adopted to determine the solid phase content of each sample. The testing procedure is as follows: weigh the sample → vacuum dry at 105 °C to constant weight → weigh again. The calculation formula for solid phase content is as follows: solid phase content = (mass of dried solid/total mass of sample before drying) × 100%. Subsequently, the relative standard deviation is calculated using the following formula: RSD = (S/μ) × 100%, where S is the standard deviation of the solid phase content at each sampling point, and μ is the mean value of the solid phase content at each sampling point. A lower RSD value indicates better uniformity of the PBX slurry.

Experimental results indicate that for the cast PBX system with 70% solid content, the kneading and mixing rate is relatively slow due to the material’s high viscosity and poor fluidity. When the kneading speed is 15 rpm and the kneading time is 3 h, the solid content test results at the three points show a large deviation from the target value of 70%. When the kneading speed is increased to 25 rpm, while the kneading time is maintained at 3 h, the solid content at each point is close to 70%, with a maximum deviation of only 0.6%, and the kneading uniformity is significantly improved. In summary, for the cast PBX system with 70% solid content, the optimal kneading process parameters are determined as follows: kneading speed of 25 rpm and kneading time of 3 h.

(2) Study on the optimal speed matching of the vertical–horizontal two-stage screws.

(a) Conveying principle of the two-stage screws.

**Figure 18:**
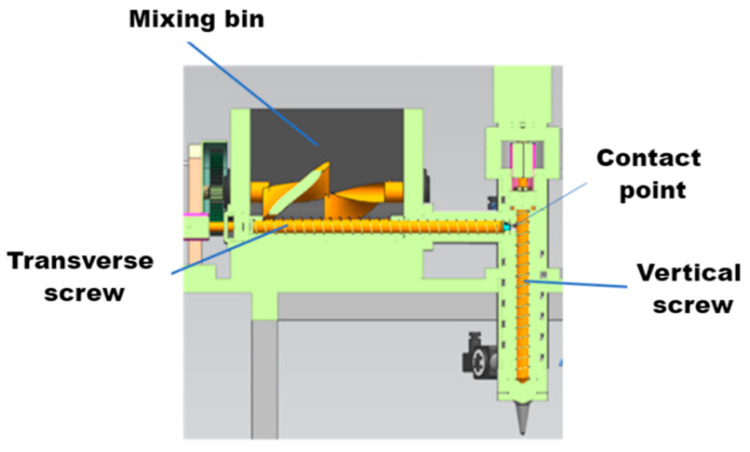
Cross-longitudinal dual-screw structure.

The vertical–horizontal two-stage screw extrusion system designed in this study undertakes the directional conveying and extrusion molding functions of energetic slurry, and its working principle is shown in Figure 18.

The horizontal screw is responsible for horizontally conveying the uniformly kneaded energetic slurry to the feeding end of the vertical screw. After receiving the material, the vertical screw vertically conveys it to the nozzle, ultimately achieving stable extrusion and molding of the slurry.

(b) Key significance of speed matching

**Figure 19:**
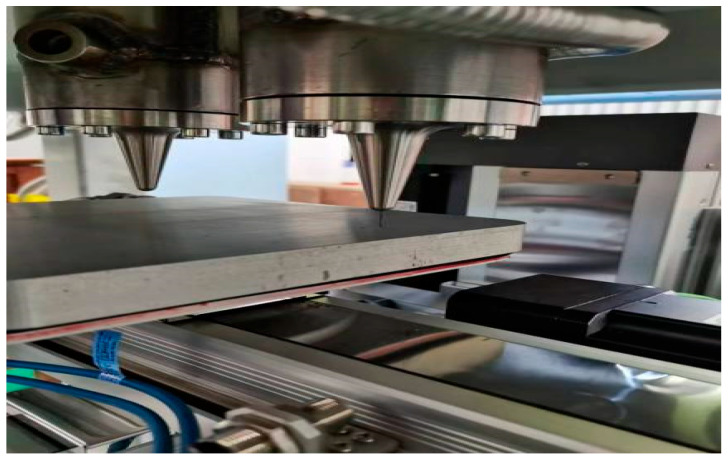
Experiment of extruding PBX into threads.

The speed matching of the two-stage screws is the core prerequisite for ensuring the conveying continuity, stability of the slurry, and operational safety of the equipment. If the conveying rate of the horizontal screw is lower than that of the vertical screw, it is prone to insufficient feeding of the vertical screw, leading to reduced conveying efficiency. If the conveying rate of the horizontal screw is higher than that of the vertical screw, materials tend to accumulate at the contact interface of the two-stage screws, causing sidewall stress concentration and flow channel blockage. This not only shortens the equipment service life but also significantly reduces production efficiency. Therefore, this study conducts research on the optimal speed matching of the kneading rate, horizontal screw conveying rate, and vertical screw extrusion rate, and the experimental process is shown in Figure 19.

(c) Experimental design and determination of optimal parameters

To obtain the optimal extrusion molding parameters, we conducted energetic slurry extrusion experiments with nozzle diameter and vertical–horizontal two-stage screw speeds as variables, and the results are shown in Table 6.

Experimental analysis indicates that the optimal speed matching relationship of the two-stage screws is achieved when the horizontal screw speed is slightly lower than the vertical screw speed. This “starved feeding mode” can effectively avoid material accumulation or insufficient feeding, enabling continuous and stable filament formation of the slurry. In addition, there is an adaptive relationship between nozzle size and screw speed: the larger the nozzle diameter, the higher the required vertical–horizontal screw speeds. Otherwise, problems such as slow discharge rate and filament stretching fracture are prone to occur. The stable filament extrusion parameters for all experimental groups must be matched to the nozzle diameter: In Group 12, the parameter combination of 10 rpm for the horizontal screw and 12 rpm for the vertical screw enables stable filament extrusion; however, after a comprehensive evaluation of filament forming accuracy and equipment energy consumption, the optimal parameters corresponding to a 1.55 mm nozzle diameter are determined as 5 rpm for the horizontal screw and 7 rpm for the vertical screw.

Based on the experimental results, the optimal equipment parameters and printing process parameters are finally determined as follows: nozzle diameter of 1.55 mm, kneading speed of 25 rpm, horizontal conveying speed of 5 rpm, vertical extrusion speed of 7 rpm, and feed rate multiplier of 100%. The ultraviolet (UV) curing process is adopted during the molding process, with the UV light power set to 15 W.

(d) Analysis of printed filament dimensions and path optimization

**Figure 20:**
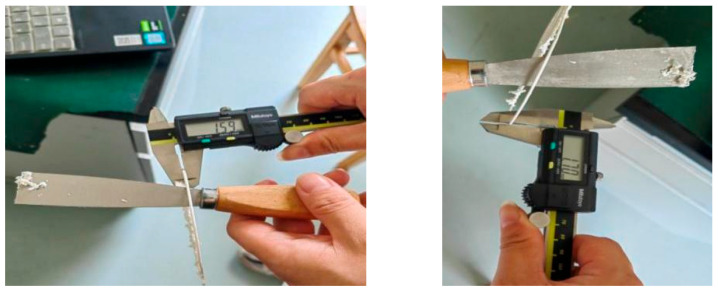
Measurement of the thickness of a printed single-layer circle.

To verify the printing quality, a single-circle printing experiment was first conducted, and the dimensions of a single cured filament were characterized (results shown in Figure 20). Tests show that the actual height and thickness of the filament extruded by the 1.55 mm nozzle are approximately 1.59 mm and 1.70 mm, respectively, both slightly higher than the nozzle diameter. This is mainly attributed to two factors: first, the “die swell effect” during slurry extrusion leads to a slight increase in the cross-sectional size of the material; second, to ensure the adhesion between the slurry and the printing platform, the distance between the nozzle and the platform is set to slightly less than 1.5 mm, and the slurry thickness is further slightly increased by the extrusion force of the nozzle during extrusion. Combined with the actual dimensional characteristics of the extruded filament, the slicing path for printing and molding will be optimized in subsequent studies to improve the molding accuracy.

**Figure 21:**
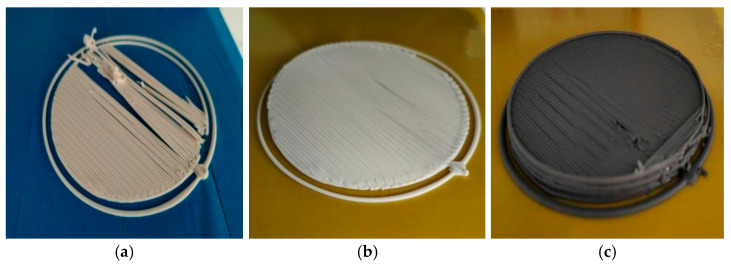
Results of single-layer printing experiment. (**a**) Single-layer printing scratch phenomenon; (**b**) Optimized single-layer printed sample; (**c**) Aluminum-containing formula medicinal paste printing sample.

Based on the above-determined optimal equipment parameters and printing process, single-layer printing verification experiments were conducted. The initial experiment adopted a 15 W UV curing process, and the printing results are shown in Figure 21a. It was found that due to the excessively high UV light power, the curing rate of the energetic slurry was too fast. The slurry not yet fully extruded at the nozzle was cured in advance during the printing process, causing scraping between the nozzle and the cured material, which affected the molding effect.

To address this issue, the UV light power was adjusted to below 10 W, and the single-layer printing experiment was repeated. The molding effect is shown in Figure 21b. Results indicate that after reducing the UV light power, the slurry curing rate matched the extrusion rate, enabling the printing process to proceed smoothly. The single-layer formed part had a flat surface without scraping, defects, or other issues, verifying the rationality of the curing process parameters.

To expand the applicability of the material system, the formulation of the energetic slurry was further adjusted by introducing Al powder as a functional component, and single-layer printing experiments were carried out (results shown in Figure 21c). Experiments demonstrate that stable stacking and molding of the energetic slurry can be achieved using a slicing printing path with outer circles and inner straight lines. However, during the layer-switching printing process, the problem of slurry residue still exists, which needs to be solved by further optimizing the extrusion rate and path switching timing.

**Figure 22:**
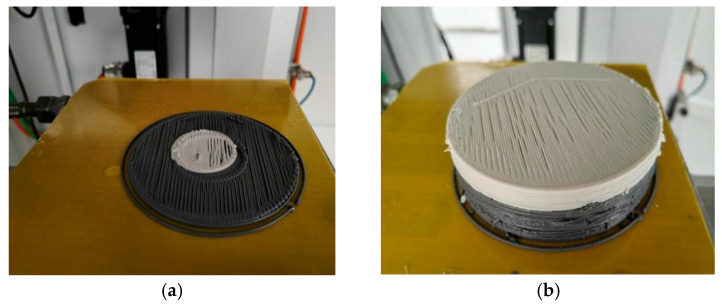
Double-nozzle printing experiment. (**a**) The double-head printing situation at different centers; (**b**) A longitudinal composite printing sample with dimensions of 100 mm by 30 mm.

To verify the coordinated molding capability and printing center consistency of the dual-nozzle mechanism, this study conducted dual-nozzle printing process experiments. First, the alignment accuracy of the dual-nozzle printing centers was tested, and the experimental results are shown in Figure 22a. There was a significant deviation between the printing center regions of the white slurry and black slurry, which affected the molding accuracy of the multi-component composite structure.

To address this issue, precise calibration of the central positions of the two nozzle mechanisms was performed. After calibration, the printing experiment was repeated, and the results are shown in Figure 22b. Experiments demonstrate that after central position calibration, the component positioning accuracy of dual-nozzle printing is significantly improved, and the preliminary realization of the multi-layer three-dimensional composite structure via dual-nozzle printing is achieved successfully. This provides process support for the coordinated molding of multi-component energetic materials.

**Figure 23:**
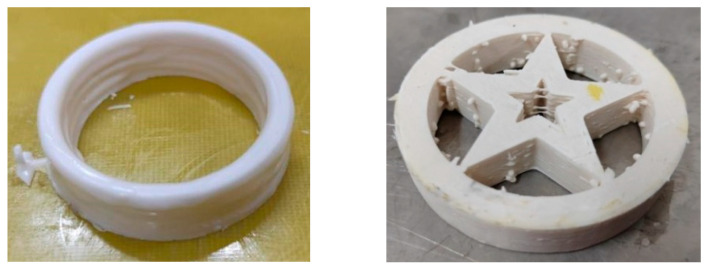
Hollow circle structure modeling and printed sample.

To further verify the molding adaptability of the developed additive manufacturing device for complex structures, this study conducted printing experiments on PBX grains with various complex special-shaped structures using the device. Typical special-shaped parts such as hollow circular structures and internal pentagram structures were successfully fabricated, and the molding results are shown in Figure 23.

Experimental results indicate that the additive manufacturing device for high-viscosity energetic materials can achieve precise molding of complex special-shaped structures, effectively verifying the structural rationality and process feasibility of the device. This provides key technical support and practical experience reference for the expansion and optimization of the additive manufacturing process for energetic materials.

## 5. Conclusions

To address the core technical challenges in the additive manufacturing of high-viscosity, high-solid-content multi-component energetic materials (polymer-bonded explosive, PBX), such as low extrusion efficiency and frequent nozzle clogging, this study systematically optimized the screw structure, process parameters, and equipment design through a combined numerical simulation and experimental verification approach, establishing a complete technical system. The main conclusions are as follows:

- Screw Structure Optimization: By comparing the pressure fields and shear rate fields of tapered and cylindrical screws, the cylindrical screw with a 3 mm clearance was determined as the optimal solution. Its minimum value of maximum pressure (2.36 MPa) is significantly lower than that of the tapered screw (3.39 MPa), with the pressure fluctuation amplitude reduced by 30%. At a 1.5 mm clearance, its maximum shear rate (937.3 s^−1^) is only 36% of that of the tapered screw (2602 s^−1^), which is more compliant with the safety threshold requirements of energetic materials. Additionally, the constant clearance design avoids the intensified backflow of the tapered screw caused by diameter variation, thereby improving the stability of material conveying.
- Process Parameter Optimization: The optimal kneading and extrusion parameters were clarified. When the Z-shaped stirring paddle operates at a kneading speed of 25 rpm for 3 h, the solid content of the slurry is close to 70%, with a maximum deviation of only 0.6% and optimal uniformity (RSD = 0.89%). During extrusion, a 1.55 mm nozzle diameter combined with a horizontal screw (feeding) speed of 5 rpm and a vertical screw (extrusion) speed of 7 rpm in the “starved feeding” mode enables continuous and stable filament formation of the slurry. This controls the system pressure fluctuation within ±0.3 MPa.
- Equipment and Molding Verification: A dual-nozzle, horizontal–vertical two-stage screw extrusion equipment was designed, integrating explosion-proof, precise temperature control, and a three-dimensional motion platform. Successful molding of complex-shaped PBX charges (e.g., hollow circular structures and internal five-pointed star structures) was achieved, verifying the equipment’s advantages in safety control, precision control, and adaptability to complex structures. This lays a theoretical and technical foundation for the engineering application of additive manufacturing for energetic materials.

## 6. Discussion

- Verification of Model Applicability: The Herschel–Bulkley–Papanastasiou (HBP) model can effectively describe the non-Newtonian fluid characteristics (yield stress, shear-thinning) of energetic slurries. Its parameters (e.g., τy = 250 Pa; m = 5.0 s) were obtained by fitting the rheological experimental data, which are highly consistent with the simulation and experimental results. The conclusion predicted by the Bingham model that “shear-thinning dominates the flow” was also verified by experiments, confirming the applicability of both models in the extrusion simulation of high-solid-content energetic materials.
- Analysis of Key Influence Mechanisms: When the horizontal screw speed is greater than or equal to the vertical screw speed, material accumulation is likely to cause flow channel blockage and a sudden increase in extrusion pressure. However, the matching relationship of “horizontal speed slightly lower than vertical speed” can avoid this problem, essentially balancing the material conveying rate and extrusion rate. The higher pressure increase slope of the high-solid-content slurry stems from enhanced energy dissipation caused by increased collision and friction between particles, providing a theoretical basis for the adaptive adjustment of subsequent process parameters.
- Research Limitations: The simulation did not couple the microscopic thermal decomposition reactions of energetic materials, which may cause it to underestimate the flow resistance at high temperatures. In situ measurement technology was not used in the experiments to obtain the local velocity distribution inside the nozzle, limiting the verification of particle movement details. Additionally, slurry residue still exists during layer-switching printing, requiring further extrusion rate and path switching timing optimization.
- Future Research Directions: Subsequent studies can develop in situ flow field measurement technology for energetic materials to accurately capture the microscopic characteristics of material conveying and extrusion. A “flow heat transfer reaction” multi-field coupled simulation model should be established to improve safety prediction accuracy. Furthermore, optimizing equipment structure and process parameters to expand the composite molding capability of multi-component energetic materials will promote the industrial implementation of the technology.

## Figures and Tables

**Table 1:** Parameters of PBX materials.

Zero Shear Viscosity (η_0_/Pa.s)	Infinite Shear Viscosity (η∞/Pa.s)	Stress Relaxation Time (λ/s)	Non-Newtonian Index (n)	Melt Density (/g/cm^3^)	Yield Stress (τy)	Yield Stress Relaxation Coefficient (m)
46249	1.507	0.0607	0.59784	1.54	250	5.0

**Table 2:** Parameter table of simulation boundary conditions.

Boundary Type	Key Parameter	Value	Unit
Inlet	Volumetric Flow Rate	100	mm^3^/s
Outlet	Normal Force (fn)	0.2	N
Outlet	Exit Pressure (Converted Value)	0.106	MPa
Outlet	Tangential Force (fs)	0	N
Inner Wall of Flow Channel (at the minor diameter of the screw)	Rotational Speed	30	rpm
Inner Wall of Flow Channel (at the minor diameter of the screw)	Motion Mode	No-slip	-
Outer Wall of Flow Channel (inner surface of the barrel)	Normal Velocity (vn)	0	mm/s
Outer Wall of Flow Channel (inner surface of the barrel)	Tangential Velocity (vs)	0	mm/s

**Table 3:** Pressure data.

Serial No.	Screw Type	Screw Clearance (mm)	Characteristic Cross-Section	Average Pressure (MPa)	Pressure Standard Deviation (MPa)	Pressure Coefficient of Variation CV (%)	Pressure Range Rp (MPa)	Pressure Relative Fluctuation Degree δ_P_ (%)
1	Tapered	1.5	S_0_	65.23	4.89	7.50	18.62	8.92
2	Tapered	1.5	S_5_	58.76	3.72	6.33	14.25	8.92
3	Tapered	1.5	S_out_	52.31	2.98	5.70	11.36	8.92
4	Tapered	2.0	S_0_	3.98	0.21	5.28	0.87	6.45
5	Tapered	2.0	S_5_	3.56	0.17	4.78	0.72	6.45
6	Tapered	2.0	S_out_	3.12	0.13	4.17	0.59	6.45
7	Tapered	2.5	S_0_	3.52	0.15	4.26	0.68	5.18
8	Tapered	2.5	S_5_	3.21	0.12	3.74	0.55	5.18
9	Tapered	2.5	S_out_	2.89	0.10	3.46	0.48	5.18
10	Tapered	3.0	S_0_	3.31	0.11	3.32	0.52	4.36
11	Tapered	3.0	S_5_	3.05	0.09	2.95	0.45	4.36
12	Tapered	3.0	S_out_	2.78	0.08	2.88	0.41	4.36
13	Cylindrical	1.5	S_0_	60.15	3.12	5.19	12.87	6.75
14	Cylindrical	1.5	S_5_	54.82	2.45	4.47	10.33	6.75
15	Cylindrical	1.5	S_out_	49.68	1.89	3.80	8.46	6.75
16	Cylindrical	2.0	S_0_	5.03	0.18	3.58	0.76	4.22
17	Cylindrical	2.0	S_5_	4.56	0.14	3.07	0.62	4.22
18	Cylindrical	2.0	S_out_	4.12	0.11	2.67	0.51	4.22
19	Cylindrical	2.5	S_0_	3.15	0.09	2.86	0.43	3.18
20	Cylindrical	2.5	S_5_	2.92	0.07	2.40	0.37	3.18
21	Cylindrical	2.5	S_out_	2.68	0.06	2.24	0.32	3.18
22	Cylindrical	3.0	S_0_	2.28	0.05	2.19	0.29	2.53
23	Cylindrical	3.0	S_5_	2.15	0.04	1.86	0.25	2.53
24	Cylindrical	3.0	S_out_	2.02	0.03	1.48	0.21	2.53

**Table 4:** Pressure–shear rate synergy analysis table under different screw clearances.

Screw Type	Screw Clearance (mm)	Maximum Pressure P_max_ (MPa)	Maximum Shear Rate γ_max_ (s^−1^)	Pressure Safety Evaluation (≤5 MPa)	Shear Rate Safety Evaluation (≤3000 s^−1^)	Comprehensive Safety Adaptability
Conical screw	1.5	68.9	2602	no	yes	Unsafe
Conical screw	2.0	4.10	3161	yes	no	Unsafe
Conical screw	2.5	3.61	3754	yes	no	Unsafe
Conical screw	3.0	3.39	4016	yes	no	Unsafe
Cylindrical screw	1.5	63.02	937.3	no	yes	Unsafe
Cylindrical screw	2.0	5.27	1184	no	yes	Critically Safe
Cylindrical screw	2.5	3.23	1824	yes	yes	Safe
Cylindrical screw	3.0	2.36	2164	yes	yes	Optimally Safe

**Table 5:** Test results of homogeneity of pouring PBX mixture.

Serial No.	Rotate Speed/rpm	Time/h	Solid Content at Point 1/%	Solid Content at Point 2/%	Solid Content at Point 3/%	Mean Solid Content (μ, %)	Standard Deviation (S, %)	Relative Standard Deviation (RSD, %)
1	15	1	62.4	77.5	64.2	68.03	8.25	12.13
2	15	2	64.7	75.1	67.3	69.03	5.05	7.32
3	15	3	67.5	72.2	68.8	69.50	2.36	3.40
4	20	1	65.2	73.9	63.7	67.60	5.20	7.69
5	20	2	65.8	71.5	65.9	67.73	3.08	4.55
6	20	3	68.3	70.4	68.6	69.10	1.10	1.59
7	25	1	66.3	72.4	65.3	68.00	3.61	5.31
8	25	2	68.5	71.3	69.1	69.63	1.40	2.01
9	25	3	69.4	70.6	69.7	69.90	0.62	0.89

**Table 6:** Extrusion experiment of energetic materials.

Serial No.	Spray Head Diameter/mm	Lateral Rotational Speed/rpm	Longitudinal Rotational Speed/rpm	Continuity	Phenomenal Description
1	1	5	4.5	1 h	Over-saturation; sticky material at the nozzle; high pressure
2	1	5	5	1.5 h	Pressure is unstable and the discharge is fluctuating
3	1	5	5.5	>3 h	Stable filament extrusion; stable extrusion pressure
4	1	7	6	0.5 h	Over-saturation; sticky material at the nozzle; high pressure
5	1	7	7	1 h	Pressure is unstable and the discharge is fluctuating
6	1	7	8	>3 h	Stable filament extrusion; stable extrusion pressure
7	1.55	10	8	1 h	Over-saturation; sticky material at the nozzle; high pressure
8	1.55	10	10	2 h	Pressure is unstable and the discharge is fluctuating
9	1.55	10	12	>3 h	Stable filament extrusion; stable extrusion pressure
10	1.55	14	12	1 h	Over-saturation; sticky material at the nozzle; high pressure
11	1.55	14	14	2 h	Pressure is unstable and the discharge is fluctuating
12	1.55	14	16	>3 h	Stable filament extrusion; stable extrusion pressure

## Data Availability

The original contributions presented in this study are included in the article. Further inquiries can be directed to the corresponding author.
